# Regional Differences and Convergence of Inter-Provincial Green Total Factor Productivity in China under Technological Heterogeneity

**DOI:** 10.3390/ijerph19095688

**Published:** 2022-05-07

**Authors:** Chong Huang, Kedong Yin, Hongbo Guo, Benshuo Yang

**Affiliations:** 1 Institute of Marine Economics and Management, Shandong University of Finance and Economics, Jinan 250014, China; huangchong@sdufe.edu.cn (C.H.); yinkedong@sdufe.edu.cn (K.Y.); 2 School of Management Science and Engineering, Shandong University of Finance and Economics, Jinan 250014, China; guohongbo@mail.sdufe.edu.cn; 3 School of Economics, Ocean University of China, Qingdao 266100, China

**Keywords:** green total factor productivity, SBM-GML Index Model, cluster cutting-edge, gravity-standard deviational ellipse, convergence

## Abstract

Green development is an effective way to reconcile the main contradictions between resources, environment, and regional development. Green total factor productivity (GTFP) is an important index to measure green development; an undesirable output-oriented SBM-DEA model and GML model can be used to calculate GTFP. China’s 30 provinces (municipalities and autonomous regions) are divided into three groups: eastern, central, and western. The common frontier function and group frontier function are established, respectively, to deeply explore the temporal and spatial evolution characteristics and center of gravity shift of inter-provincial green total factor productivity (GTFP) in China, and test the convergence under group frontier, to compare the convergence problems under different regions. This study aims to point out the differences in economic growth in different regions of China, foster regional coordination and orderly progress, promote China’s green development process, and improve the high-quality economic development level. According to the results, the efficiency of green development is more reasonable under the frontier groups. The average TGR in the eastern region was 0.993, indicating that it reached 99.3% of the meta-frontier green development efficiency technology. The inter-provincial GTFP in China gradually increased, with an average value of 1.043, which means China’s green development and ecological civilization construction have achieved remarkable results and the three regions showed significant differences. Judging from the shift path of the spatial center of gravity, the spatial distribution pattern of inter-provincial GTFP in China tends to be concentrated and stable as a whole. Moreover, *σ* convergence only exists in the western region, while absolute *β* convergence and conditional *β* convergence exist in eastern, central, and western regions, indicating that the GTFP of different regions will converge to their stable states over time. The results provide a basis for improving the efficiency of institutional allocation of environmental resources, implementing regional differentiated environmental regulation policies, and increasing the value creation of factor resources, which is of great significance for realizing the high-quality economic development in which resources, environment, and economy are coordinated in China.

## 1. Introduction

In the context of the “double carbon” target, green transformation is being actively pursued as part of economic development in China. A green development target was proposed in the 2021 Report on the Work of the Government, requiring an 18% reduction in carbon dioxide (CO_2_) emissions, a 13.5% reduction in energy consumption per unit of GDP, and positive and sustained improvements in environmental quality. Ecological civilization is a social form with the fundamental purpose of harmonious coexistence, virtuous cycle, all-around development, and sustainable prosperity between man and nature. Ecological civilization is the sum of material and spiritual achievements made by human beings following the objective law of harmonious development of man, nature, and society. “Achieving new progress in the construction of ecological civilization” is one of the main social and economic development goals of China during the 14th Five-Year Plan period (2021–2025). Given China’s gradual high-quality economic development, the environment and energy have had a restrictive impact on society and the economy. Green total factor productivity (GTFP) incorporates factors such as environmental pollution and energy consumption based on traditional total factor productivity (TFP). Thus, GTFP is more in line with the current target planning of high-quality economic development proposed by China. GTFP incorporates various factors, such as energy and resources, into the TFP framework, which can realize the decoupling of economic growth from resource consumption and pollution emissions and is an important indicator to measure the coordinated development of resources, environment, and economy of a country or region. Since the 18th National Congress of the Chinese Communist Party (CCP), China has placed the construction of the ecological civilization in a very prominent position to achieve green and orderly social and economic progress. In addition, the 19th National Congress of the CCP has emphasized promoting the construction of an ecological civilization system. Therefore, for managing resource and environmental constraints, enhancing the level of GTFP has become one of the mainstream trends in China. By analyzing the spatial and temporal evolution characteristics of GTFP in different regions, the convergence test identifies the differences in regional economic growth, enhances regional coordination and orderly advancement, and promotes the process of green development in China.

Experts and scholars outside China began studying TFP at an early stage. Academic circles generally believe that Solow (1957) is the pioneer of the theory. In particular, the “Solow residual” theory was one of the foundations of later research [1]. Farrell (1957) used linear programming to solve the problem of possible technical inefficiency of producers. Considering that not all producers can be located at the frontier of the production function, they were measured in both parametric and non-parametric ways [2]. Following Farrell’s multiple-input, multiple-output model, Charnes et al. (1978) built on this research to innovate data envelopment analysis (DEA), used in operations research and economic production to broadly measure production efficiency. However, these classical theories did not incorporate the impact of the environment, resources, and other factors in the input factors of economic growth. In the wake of increasingly acute environmental issues, the ecological environment, energy consumption, and other factors have been added to the research on productivity factors [3]. Chung (1997) pointed out that pollution emissions are undesired output for economic growth, giving birth to the GTFP analysis [4]. Dettori et al. (2012) studied intangible assets, spatial dependence, and total factor productivity in European regions, which shows that intangible assets are the main reason for the change in total factor productivity in Europe [5]. Moghaddasi et al. (2017) used the Solow residual method to study the relationship between Iran’s energy consumption and agricultural total factor productivity (TFP) growth from 1974 to 2012 [6]. Coomes et al. (2019). empirically studied the dynamic interaction between total factor productivity growth, agricultural system sustainability, and resilience [7]. Shair et al. (2021) analyzed the relationship between GTP growth and the efficiency of the Pakistani banking industry. The results show that the correlation between environment and banking development is negative [8]. Compared with the traditional total factor productivity, it is more scientific to include GTFP in measuring the quality of economic growth. Scholars have successively proposed GTFP that considers energy consumption and pollution emission [9,10,11].

GTFP is a new definition of environmental and energy constraints [12]. Pollutants as unexpected output are the idea of many scholars to calculate GTFP [13,14]. Umetsu et al. (2013) studied the regional differences in rice industry efficiency, total factor productivity, and technological change in the post-green period in the Philippines [15]. Wang et al. (2018) used green GDP as total output and sulfur dioxide emissions as undesired outputs to obtain green TFP [16]. Ahmed et al. (2018) pointed out that green productivity embodies the concept of sustainable development of technological progress [17]. Xu et al. (2019) estimated the agricultural development efficiency, which considers agricultural carbon emissions as an undesired output, and constructs the global Malmquist–Luenberger index [18]. Many studies analyzed data from different countries and revealed that CO_2_ emissions affect the TFP model [19,20]. Loganathan et al. (2020) investigated the effects of natural resources, green tax, and total factor productivity on a clean environment; the results show that total factor productivity needs to be improved through environmental innovation [21]. Fang et al. (2021) supplemented the determinants of agricultural GTFP; the result shows that crop insurance plays a prominent role in promoting agricultural GTFP [22]. Zhang et al. (2021) constructed a GTFP model involving technical, government, and economic [23]. Jiang (2015) investigated the linkage between China’s pollution emissions and output growth, which shows that the growth of TFP was accompanied by increasing pollution emissions [24]. Shao et al. (2016) adopted capital, labor, energy, and carbon emissions to investigate and compare the degrees of technological change [25]. Lei and Wu (2019) investigated the nonlinear effects based on the perspective of government and private regulation on GTFP; the results indicate that GTFP takes the characteristic of cyclical fluctuation [26]. Li and Li (2019) found that environmental regulation can effectively promote green industrial transformation by improving GTFP [27]. Feng et al. (2021) systematically analyzed the impact of FDI, OFDI, and their interaction on China’s GTFP using a spatial econometric model [28]. Zhou et al. (2021) empirically tested the effect and mechanism of digital economy development on GTFP based on the panel data of Chinese cities from 2011 to 2019 [29]. Khan et al. (2021) pointed out that green total factor productivity is the measure of a country or region’s ability to achieve long-term sustainable development goals [30]. Zhao (2022) analyzed the evolutionary characteristics and interactive relationship between industrial transformation and upgrading and the promotion of GTFP [31]. Li et al. (2022) analyzed the relationship between innovation spillover capacity, GTFP, and temporal and spatial patterns of 30 provinces in China from 2008 to 2018 from two dimensions of time and space [32].

DEA has been widely used in empirical analyses [33,34]. Li et al. (2009) proposed the meaning of GTFP and incorporated variables reflecting environmental fluctuations in the TFP calculation [35]. Chen (2009) applied the transcendental logarithm to account for the green growth of industrial TFP fluctuations and analyzed how to promote the sustainable green development of China’s industry [36]. Li et al. (2013) applied the ML productivity index and slack-based measure (SBM) efficiency measurement model to analyze the GTFP of sub-industries in China [37]. Chen et al. (2016) identified the corresponding explanatory variables in a way that transcended the logarithmic production function and applied the empirical analysis model to finally obtain GFTP [38]. Li et al. (2018) adopted the non-radial directional distance function to analyze green economic growth evaluation index construction [39]. Based on the Green Development Indicator System, Wang et al. (2018) [40] evaluated the green development statuses of 30 provinces in China. The spatial autocorrelation and convergence models were used to analyze China’s evolution characteristics and spatial pattern of green development. Li and Liu (2019) analyzed the economic growth clusters in the Yangtze River Delta, Pearl River Delta, and Beijing–Tianjin–Hebei regions in China through an inefficiency model and found that resources and environment were the main factors leading to the GTFP inefficiency [41]. Amani et al. (2019) considered the use of relaxation-based measurement (SBM) model transformation index in variable return on Scale (VRS) to develop MPI [42]. Balezentis et al. (2020) used the environmental Luenberger-Hicks-Moorsteen productivity index based on the distance function of input and output directions to measure the green economic growth of the agricultural sector in selected European countries [43]. Teng et al. (2020) measured the GTFP of China’s service industry using the SBM efficiency model and global ML index for the first time and adopted the Gini coefficient to study the degree and source of its spatial divergence. Next, the driving factors of spatial divergence were examined [44]. Wu et al. (2020) used the DEA-GML index to measure GTFP in 30 provinces (cities) of China and conducted a study on the variability of the impact of environmental regulation from the perspective of GTFP [45]. Lin et al. (2020) used panel data of the Beijing–Tianjin–Hebei city cluster to construct a transcendental logarithmic production function SFA (suitability, feasibility, and acceptability) model to empirically assess and analytically deconstruct the quality and motive force of economic development based on GTFP [46]. Li et al. (2021) used the fixed-effect model and threshold regression model to empirically investigate the internal mechanism and influence intensity of GTFP in areas affected by Internet development [47]. Song et al. (2022) found that environmental regulation “forces” the green technology progress of enterprises through external pressure, which significantly promotes the TFP of enterprises [48].

Drawing upon the foundation and research findings of previous studies, this study empirically examined the GTFP of 30 provinces (municipalities and autonomous regions) in China under the dual constraints of energy consumption and environmental pollution. It analyzed the inter-provincial GTFP spatio-temporal evolution, regional differences, and spatial center of gravity shift in China in the establishment of group frontier and meta-frontier functions. Thereafter, this paper explored σ convergence under group frontier, absolute β, and conditional β convergence of GTFP to identify the spatial and temporal variation characteristics of GTFP and provide feasible suggestions for realizing regional green, coordinated, and high-quality economic development in China. The research of this paper can promote a more reasonable allocation of limited resources, improve the efficiency of resource allocation, balance the differences in economic growth between different regions, truly realize the transformation and upgrading of the green economy, and provide a factual basis for the transformation of China’s economy from high-speed growth to high-quality growth.

## 2. Methodology and Data

### 2.1. SBM-DEA Model

An undesirable output-oriented SBM-DEA model was used to calculate GTFP, which was proposed by Tone (2001) [49]. This model is based on non-angle and non-radial relaxation variables, has the characteristic of not changing the returns to scale, and includes non-consensual output. The SBM-DEA model for a specific decision unit takes the following form.

(1) $$\begin{array}{c} \rho =\min\frac{1-\frac{1}{I}\sum_{i=1}^{I}s_{i}^{x}/x_{k^{\prime }i}^{t^{\prime }}}{1+\frac{1}{E+U}\left(\sum_{e=1}^{E}s_{e}^{y}/y_{k^{\prime }e}^{t^{\prime }}+\sum_{u=1}^{U}s_{u}^{z}/z_{k^{\prime }u}^{t^{\prime }}\right)}\\ s.t.\;\sum_{t=1}^{T}\sum_{k=1}^{K}\lambda_{k}^{t}x_{ki}^{t}+s_{i}^{x}=x_{k^{i}}^{t^{\prime }},\;i=1,\ldots ,I\\ \sum_{t=1}^{T}\sum_{k=1}^{K}\lambda_{k}^{t}y_{ke}^{t}-s_{e}^{y}=y_{k^{\prime }e}^{t^{\prime }},\;e=1,\ldots ,E\\ \sum_{t=1}^{T}\sum_{k=1}^{K}\lambda_{k}^{t}z_{ku}^{t}+s_{u}^{z}=z_{k^{\prime }u}^{t^{\prime }},\;u=1,\ldots ,U\\ \lambda_{k}^{t}⩾0,\;s_{i}^{x}⩾0,\;s_{e}^{y}⩾0,\;s_{u}^{z}⩾0,\;k=1,\ldots ,K; \end{array}$$

where the input type is represented by *I*, and the desirable and undesirable output types are denoted by *E* and *U*, respectively. *x*, *y*, and *z* denote the input, desirable output, and undesirable output vector. $s_{i}^{x}$ refers to the input–output redundancy; $s_{u}^{z}$ denotes undesirable output redundancy; $s_{e}^{y}$ denotes the desirable output deficiency; $\left(x_{k^{\prime }i}^{t^{\prime }},y_{k^{\prime }e}^{t^{\prime }},b_{k^{\prime }u}^{t^{\prime }}\right)$ denotes the input–output value of the first $k^{\prime }$ production unit corresponding to in the $t^{\prime }$ time period. $\lambda_{k}^{t}$ represents the corresponding weight of the decision unit. The objective function ρ strictly monotonically decreases with $s_{i}^{x},s_{e}^{y},s_{u}^{z}$, while satisfying 0<ρ≤1. When ρ=1, $s_{i}^{x}=s_{e}^{y}=s_{u}^{z}=0$. Neither redundancy nor deficiency in the input–output exists, indicating that the evaluated decision unit is efficient. DEA is considered invalid when ρ<1. The input-output quantity must be deeply optimized to improve its efficiency.

### 2.2. Common and Group Frontiers

**Figure 1:**
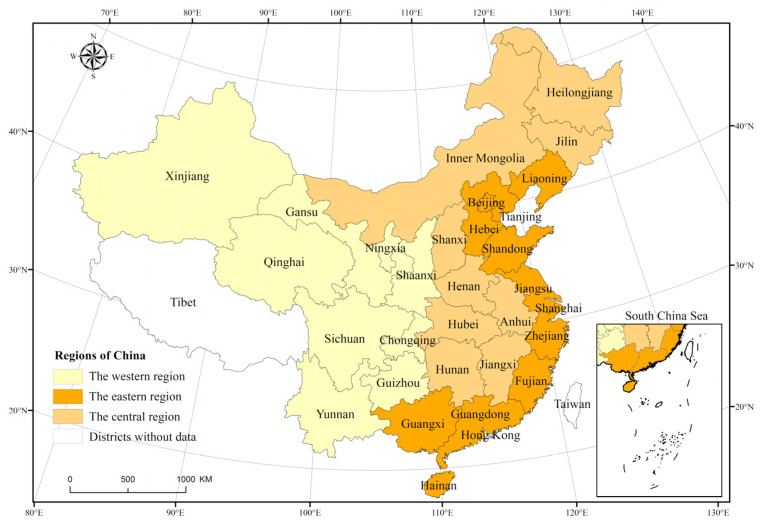
Three major clusters—east, central, and west of China.

Battese (2004) [50] was the first to propose the meanings of group-frontier and meta-frontier. Meta-frontier indicates the potential technical quality of decision-making units (DMUs). Group frontier refers to the actual technology level of different DMUs. The model analyzes the gap with the meta-frontier for grouped data, each of which has a different set of technologies referenced. According to the classification standard of the National Bureau of Statistics of China, China’s provincial administrative divisions were divided into three major clusters—East, Central, and West—to explore the efficiency of green development under the meta-frontier and group frontier, respectively (Figure 1). The division standard is reasonable.

According to the meta-frontier model proposed by Battese (2004) [50], the set of technologies under the meta-frontier ($T^{meta}$) is constructed as follows.

(2) $$T^{meta}=\left\{\begin{array}{l} \left(x,y^{e},z^{u}\right):x\geq 0,\;y^{e}\geq 0,\\ z^{u}\geq 0;x\;\mathrm{is}\;\mathrm{able}\;\mathrm{to}\;\mathrm{produce}\;\left(y^{e},z^{u}\right) \end{array}\right\},\;$$

where *x* represents the input vector, *y*^e^ denotes the desirable output vector, $z^{u}$ represents the undesirable output vector, and the meta-boundary associated with it is:

(3) $$P^{meta}\left(x\right)=\left\{\left(y^{e},z^{u}\right):\left(x,y^{e},z^{u}\right)\in T^{meta}\right\}.\;$$

The meta-distance function can be expressed as follows:

(4) $$D^{meta}\left(x\right)=\left(x,y^{e},z^{u}\right)=\sup_{\lambda }\left\{\lambda >0:\left(x/\lambda \right)\in P^{meta}\left(y^{e},z^{u}\right)\right\}.\;$$

China’s provincial administrative divisions were divided into the eastern, central, and western regions (i=1,2,3). Their technology sets can be expressed as:

(5) $$T^{group}=\left\{\left(x_{i},y_{i}^{e},z_{i}^{u}\right):x_{i}\geq 0,\;y_{i}^{e}\geq 0,\;z_{i}^{u}\geq 0;\;x_{i}\to \left(y_{i}^{e},z_{i}^{u}\right)\right\},\;i=1,2,3.\;$$

Its corresponding set of production possibilities (meta-frontier) is:

(6) $$P^{group}\left(x_{i}\right)=\left\{\left(y_{i}^{e},z_{i}^{u}\right):\left(x_{i},y_{i}^{e},z_{i}^{u}\right)\in T^{group}\right\},\;i=1,2,3.\;$$

The group distance function can be expressed as follows:

(7) $$D^{group}\left(x_{i},y_{i}^{e},z_{i}^{u}\right)=\sup_{\lambda }\left\{\lambda >0:\left(x_{i}/\lambda \right)\in P^{group}\left(y_{i}^{e},z_{i}^{b}\right)\right\},\;i=1,2,3.\;$$

The $T^{meta}$ covers all group technologies, that is, $T^{meta}=\left\{T_{1}^{group}UT_{2}^{group}UT_{3}^{group}\right\}$.

### 2.3. Technological Gap Ratio

The technological gap ratio (TGR) measures the gap between meta-frontier efficiency and group frontier efficiency and provides insight into the technical heterogeneity of green development efficiency among different groups. Since the value of meta-frontier efficiency is less than or equal to the value of group frontier efficiency, the TGR takes a range of [0, 1]. A higher TGR value indicates that the actual group production efficiency is closer to the potential production efficiency. On the contrary, a lower TGR value indicates that the actual group production efficiency is farther from the potential production efficiency. In addition, TGR can evaluate and analyze group division. It is necessary to carry out grouping in case of a low mean TGR. A high mean TGR shows a low necessity to perform grouping.

TGR can be expressed using the meta-distance function and group distance function.

(8) $$TGR\left(x_{i},y_{i}^{e},z_{i}^{u}\right)=\frac{TE^{meta}\left(x,y^{e},z^{b}\right)}{TE^{group}\left(x_{i},y_{i}^{e},z_{i}^{b}\right)}=\frac{D^{group}\left(x_{i},y_{i}^{e},z_{i}^{u}\right)}{D^{meta}\left(x,y^{e},z^{u}\right)},\;i=1,2,3$$

### 2.4. GML Index

The traditional Malmquist Total Factor Productivity Index model may be non-transmissible or even non-solvable. This study drew on the research by Oh (2010) [51] to construct a Global-Malmquist–Luenberger (GML) index based on the SBM directional distance function to measure inter-provincial GTFP. The specific formula is as follows.

(9) $$GML_{t}^{t+1}=\frac{1+{\vec{S}}_{V}^{G}\left(x^{t},y^{t},z^{t};g^{x},g^{y},g^{z}\right)}{1+{\vec{S}}_{V}^{G}\left(x^{t+1},y^{t+1},z^{t+1};g^{x},g^{y},g^{z}\right)},\;$$

where ${\vec{S}}_{V}^{G}\left(x^{t},y^{t},z^{t};g^{x},g^{y},g^{z}\right)$ index represents the current and global SBM directional distance function constructed on non-radial and non-angular measurement methods. GML index refers to the change of the t+1 period relative to t the period. If the index is greater than 1, it implies that GTFP shows an increasing trend. If the index is less than 1, it means that GTFP shows a decreasing trend. If the index is equal to 1, GTFP is in a stable state.

### 2.5. Selection of Indicators and Data Processing

#### 2.5.1. Indicators for GTFP Measurement

Input indicators and desirable output indicators: Among them, labor input was used as a proxy for the workforce size in different provinces. The “perpetual inventory method” [52] was used for calculating the capital stock indicator. The corresponding formula is:

(10) $$K_{i,t}=I_{i,t}+(1-\delta_{i,t})K_{i,t-1}$$

where $K_{i,t}$ denotes the capital stock size of the region *i* in the year *t*. $I_{i,t}$ denotes the investment size of the region *i* in the year *t*. $\delta_{i,t}$ represents the level of depreciation rate of fixed assets in the year *t*. In line with existing research [53,54], we selected the depreciation rate of 10.96%. The total regional energy consumption converted into standard coal was selected as the energy input index. GDP deflator was used to calculate the actual GDP of each region as the expected output indicator with the year 2000 as the base period.

Non-desirable output indicators: Based on the main control targets proposed by the energy conservation and emission reduction program in China’s 13th Five-Year Plan, and considering that China’s current environmental constraint mechanism cannot be fully reflected by one pollutant alone, the non-desirable outputs were selected from CO_2_ emissions, industrial waste gas, wastewater, and general solid waste emissions of each province. Among them, CO_2_ emissions were obtained from carbon accounting databases.

#### 2.5.2. GTFP Impact Indicators

We selected four other indicators as controls for GTFP: industry structure (Industry), fiscal concentration (Finance), openness to the outside world (Open), and energy consumption structure (Energy). The share of tertiary industry output in GDP represents the industry structure. The share of regional fiscal expenditure in GDP represents the fiscal concentration. The measure of openness is the ratio of regional exports and imports to GDP. Energy consumption structure is measured by the share of natural gas consumption in total regional energy consumption.

## 3. Analysis of Inter-Provincial GTFP Measurement in China

### 3.1. Data Sources and Descriptive Statistics

Given that available statistics on non-desirable output indicators have been published only up to 2017, and to maintain data consistency, we selected panel data corresponding to 30 provinces (municipalities and autonomous regions) across the country between 2001 and 2017. The results of the descriptive statistics are presented in Table 1.

### 3.2. Comparative Analysis of China’s Inter-Provincial Green Development Efficiency under Meta-Frontier and Group Frontier

**Figure 2:**
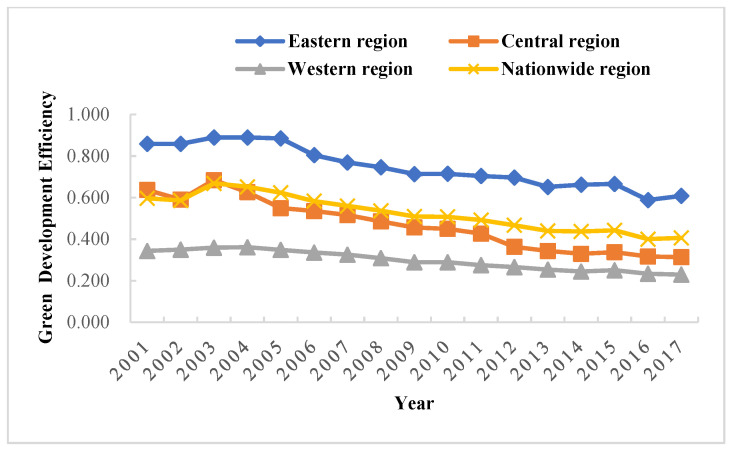
Green development efficiency under the meta-frontier of east, central, and west China.

**Figure 3:**
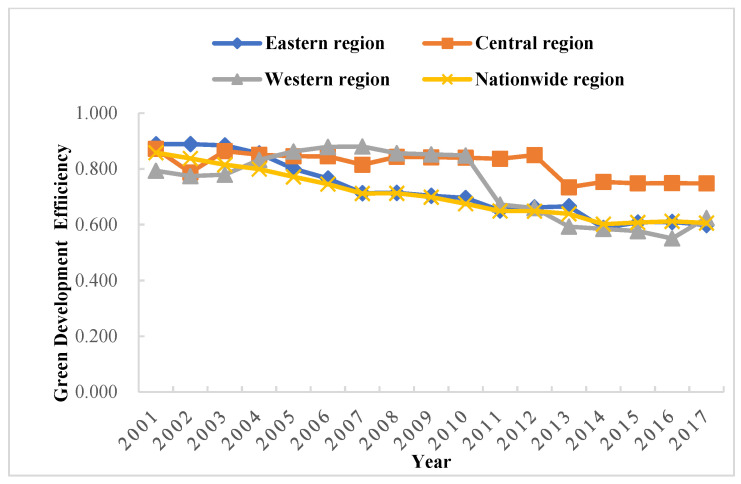
Green development efficiency under the group frontier of east, central, and west China.

**Figure 4:**
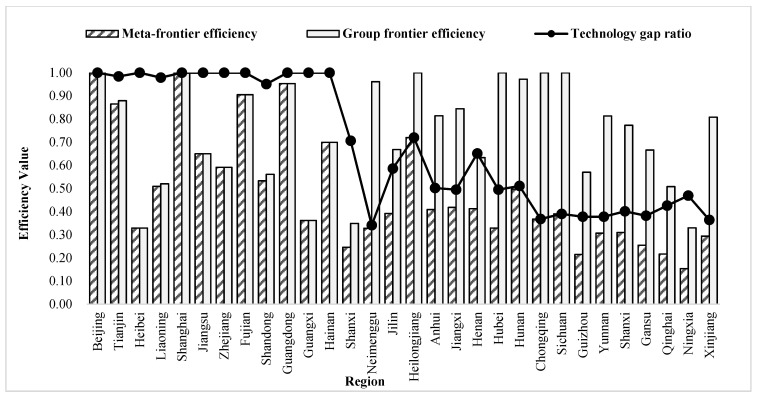
Comparison of China’s inter-provincial green development efficiency under meta-frontier and group frontier from 2001–2017.

The non-radial SBM-DEA model was used to measure the green development efficiency (Figure 2 and Figure 3) under the meta-frontier and group frontier of three major groups in China from 2001 to 2017, respectively, and the green development efficiency technology gap ratio between them (Table 2 and Figure 4). Under the meta-frontier, during 2001–2017, the mean values of inter-provincial GTFP in China were from the east region (0.657), the central region (0.400), and the west region (0.269), with each region showing a slow decline. Large differences were observed in the central and western regions compared with the eastern region, indicating room for optimization in the central and western regions. Meanwhile, with respect to the group frontier, the central region (0.770) had the highest mean value of inter-provincial green development efficiency, followed by the west (0.683) and the east (0.662). By comparing the common and group frontier results, the eastern region showed little change in efficiency values due to its proximity to the meta-frontier surface. In contrast, the central region increased from 0.440 to 0.700, and the west region increased from 0.269 to 0.683 in efficiency values under their new frontiers, which displayed a substantial increase, as DMUs with the highest efficiency within their respective clusters constitute the new frontier envelopes.

The average TGR in the eastern region was 0.993, indicating that it reached 99.3% of the meta-frontier green development efficiency technology. In particular, the wide application of energy-saving technology led to a significant increase in green development efficiency in the eastern region. In addition, factors such as the management level and institutional optimization have also contributed to the technology in the eastern region being closer to the meta-frontier. TGR in the central and western regions were 54.4% and 39.4%, respectively. In the meta-frontier context, the optimizable margin was 34.3% in the eastern, 60% in the central, and 73.1% in the western region. Accordingly, with respect to the group-frontier context, the optimizable margin was 33.8% in the eastern, 33% in the central, and 31.7% in the western region. The efficiency of green development was significantly enhanced under the group-frontier.

In terms of the green development efficiency of provinces, Beijing and Shanghai in the east outperformed the others. The corresponding mean green development efficiency value was 1, both in the group-frontier and meta-frontier contexts. Meanwhile, Tianjin, Fujian, and Guangdong regions had a mean value greater than 0.8 in both frontiers, belonging to a relatively efficient zone for green development efficiency. Due to their predominant geographic location, these cities have established a solid foundation and pay attention to environmental protection and the optimal allocation of resources. Hebei performed the worst in the eastern region, with a mean value of 0.329 under the group-frontier and meta-frontier contexts. Shanxi performed the worst in the central region, with meta-frontier and group-frontier mean values of 0.246 and 0.349. Ningxia was the worst performer in both contexts in the western region, with room for improvement in green development efficiency of 84.6% and 77%, respectively. Hubei, Shanxi, and Ningxia were “hardest hit” by environmental pollution in China’s central and western regions. In these regions, social benefits and environmental protection were neglected in the pursuit of economic development, resulting in low green development efficiency. Broadly, a large development gap was observed among various regions in China, with a significant phenomenon of “strong in the east and weak in the west.” The central and western regions need to be continuously optimized in terms of both support capacity and development environment.

In summary, there were large differences in green development efficiency under the group frontier and meta-frontier in the eastern, central, and western regions. The underlying reason was the large technology gap in China’s 30 provinces relative to different technology frontiers, thus contributing to a large difference in the level of green development efficiency using technology. Only some provinces in each region corresponded to a mean value of 1 in green development efficiency. On the one hand, this is mainly because these areas are the pioneer areas of China’s reforms and opening up, with a high level of economic development and pay more attention to the rational allocation of resources and green development. On the other hand, the green development efficiency value in other provinces was mostly below 0.5 due to the excessive pursuit of economic benefits and the relative neglect of environmental and social benefits in the development process of these areas. The combination of these reasons leads to the difference in green efficiency in different regions. Hence, when considering development in the future, it would be necessary to take measures, such as improving all regions of China’s green development efficiency and resource allocation system and enhancing technological innovation.

### 3.3. Characteristics of the Changing Spatial Distribution Pattern of GTFP between Provinces in China under the Group Frontier

#### 3.3.1. Evolution of the Spatial Pattern of Inter-Provincial GTFP in China

China’s inter-provincial GTFP under the group frontier was measured by the GML index based on the SBM directional distance function. In 2002, the 16th CPC National Congress of China proposed the development of ecological civilization. In 2008, the third meeting of the CPC Central Committee of China proposed that the construction of ecological civilization is the goal of realizing a well-off society. In 2013, the Third Plenary Session of the 18th CPC Central Committee of China incorporated the construction of ecological civilization into the five-in-one overall layout of the cause of socialism with Chinese characteristics. In 2017, the report of the 19th National Congress of the Communist Party of China listed “ecological civilization” as an important part of the new journey of socialist modernization. The results of important years are shown in Table 3.

**Figure 5:**
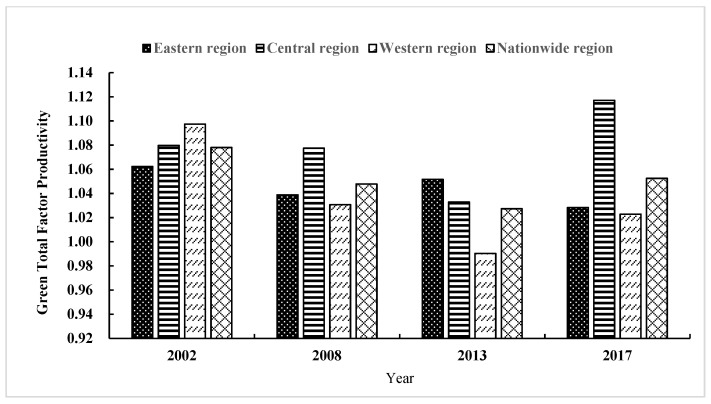
Changing trends in green total factor productivity (GTFP) by region.

Table 3 and Figure 5 demonstrate the time distribution of GTFP and its changing trend in 2002, 2008, 2013, and 2017. GTFP has evident phase characteristics. The average value of GTFP was 1.043, indicating that under the environmental and energy constraints, GTFP has gradually increased. In addition, China has switched from an extensive and low-quality economic growth model to an intensive and efficient one. From the time-dimension perspective, GTFP displayed a decreasing trend from 2002 to 2013 and gradually increased and stabilized after 2013. From the regional analysis perspective, the three regions of China indicated significant differences. The western region remained consistent with the nationwide trend, showing a “downward–upward” trend. The overall development of GTFP in the eastern region was relatively stable, while GTFP in the central region displayed an upward trend. It was evident that the central region enhanced its GTFP by continuously improving innovation technology and science and technology.

China’s inter-provincial GTFP has varied between regions from 2002 to 2017, with a downward trend in overall efficiency, indicating that GTFP was still in need of improvement. In 2002, provinces and municipalities in the eastern region with higher GTFP included Beijing, Tianjin, Liaoning, Shanghai, Jiangsu, Guangdong, and Shandong. Provinces in the central region with higher GTFP were Heilongjiang, Anhui, Hunan, Henan, and Hubei. Provinces in the western region with higher GTFP encompassed Xinjiang, Chongqing, Sichuan, Qinghai, and Yunnan. The year 2017 witnessed a substantial decline in GTFP in Liaoning, Jilin, Heilongjiang, Guangdong, Chongqing, Guizhou, and Shaanxi indicating the weakening capability of creating economic output from factors such as labor, capital, and energy. In addition, amid resource and environmental constraints, the emission of industrial waste exerted a great negative impact on the increasing GTFP. During the last 17 years, regions with low GTFP included Hebei, Hainan, Shanxi, Anhui, Jiangxi, Shaanxi, and Guizhou, all of which had room for substantial enhancement. The central and western regions focused on continuous improvement of their innovative technology and management to ensure that GTFP is maintained in a reasonable and scientific state.

**Figure 6:**
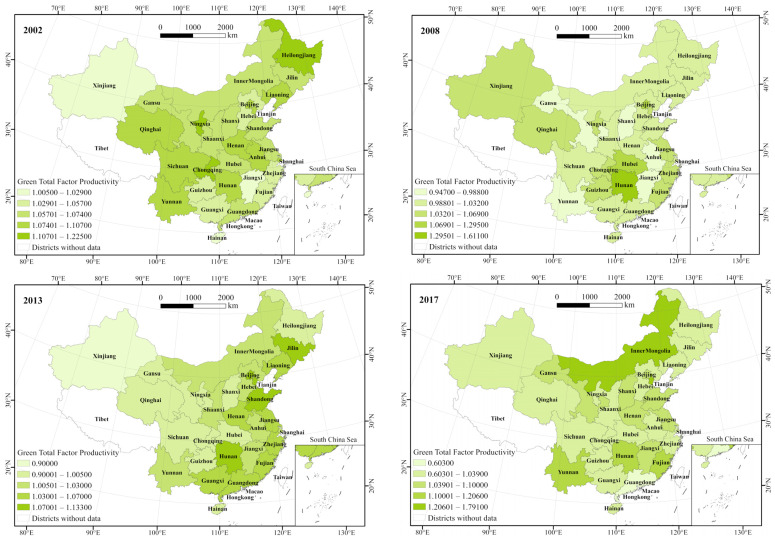
Evolution of the spatial pattern of inter-provincial GTFP in China.

Figure 6 illustrates the spatial distribution of GTFP in China’s 30 provinces (municipalities and autonomous regions) in 2002, 2008, 2013, and 2017. Based on the empirical results, GTFP values were divided into four intervals and were proportional to color shades.

#### 3.3.2. Inter-Provincial GTFP Gravity Center Shifts Path in China

Based on GTFP calculated under the group frontier, the parameters related to the center of gravity-standard deviational ellipse and the spatial location transfer path of China’s inter-provincial GTFP during 2002–2017 were analyzed. The center of gravity of efficiency can reflect the spatial distribution of the intensive utilization of GTFP, while the standard deviational ellipse can reflect the spatial dispersion degree of GTFP distribution.

**Figure 7:**
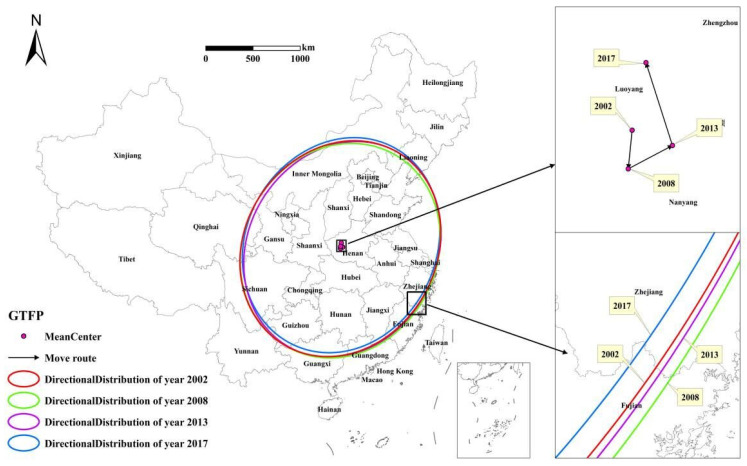
Inter-provincial GTFP standard deviational ellipse and center of gravity shift path in China.

Table 4 and Figure 7 show the distribution and movement of the GTFP’s center of gravity. The center of gravity of China’s inter-provincial GTFP shifted from 112.14° E to 112.44° E and 33.95° N to 34.45° N from 2002 to 2017, indicating the spatial stability of the GTFP during the study period. Combined with the time series, from 2002 to 2008, in China, the GTFP’s center of gravity displayed a trend of shifting toward the southwest direction, with a moving distance of 21.94 km. From 2008 to 2013, the center of gravity of GTFP showed a shifting trend toward the northeast direction, with a moving distance of 30.27 km. From 2013 to 2017, the center of gravity continued to shift toward the northwest direction, with a moving distance of 10.85 km. The moving distance of the center of gravity increased in the early study period (2002–2013) and began to shrink significantly in the later study period. From 2002 to 2017, the center of gravity of GTFP shifted in the east-west direction. Its shifting speed first increased and slowly decreased, from 1.57 km/a in 2008 to 5.25 km/a in 2013, and then decreased to 2.04 km/a in 2017. The shifting speed of the center of gravity indicated a slowly decreasing trend in the north–south direction.

#### 3.3.3. Standard Deviational Ellipsoid Analysis of Inter-Provincial GTFP in China

From the standard deviational ellipse parameters (Table 5), the range of variation of the long semi-axis of the ellipse from 2002 to 2017 was 1160.909 to 1180.737 km, while the range of variation of the short semi-axis was from 1019.724 to 1045.959 km. The standard deviational ellipse rotation angle was from 40.826° to 44.139°. The trend of the standard deviational ellipse range gradually decreased at each characteristic time point, indicating that the spatial distribution pattern of inter-provincial GTFP in China tended to be more and more concentrated. The shape index of the standard deviational ellipse was consistent with the change in the area of the standard deviational ellipse. The shape index kept decreasing, except in 2008, when it increased and displayed a trend of deviation from the positive circle, indicating that China’s inter-provincial GTFP was out of balance in east–west and north–south directions. Specifically, in the early period of the study (2002–2008), the long semi-axis shortened from 1180.737 km in 2002 to 1160.909 km in 2008, while the short semi-axis length extended from 1036.576 km in 2002 to 1045.959 km in 2008. In addition, the elliptical rotation angle kept decreasing, and the shape index of standard deviational ellipse had an expanding trend, increasingly changing to positive circles. In the middle and later stages of the study (2008–2017), the length of the long semi-axis first increased and then decreased. The length of the short semi-axis first decreased and then gradually increased. The elliptical rotation angle fluctuated less with a gradual reduction trend, revealing that the spatial distribution pattern of inter-provincial GTFP in China was relatively stable.

### 3.4. Inter-Provincial GTFP Convergence Analysis in China under Group Frontier

To enable a better in-depth analysis of different regions of inter-provincial GTFP, this section describes the convergence tests and explores the characteristics of regional differences in GTFP and their causes more deeply. The σ convergence is mainly used to reflect the difference in the degree of deviation between a region and the overall average development level and the resulting dynamic evolutionary trend. Nevertheless, it cannot show the transferability effect between different regions. Absolute β convergence refers to the gradual convergence to a slow state of variability among different variables over time, regardless of the existence of differences in the respective structural characteristics of economies. Conditional β convergence refers to the existence of similarity in terms of structural characteristics of economies and the gradual reduction of variability among different variables over time; in other words, the study of whether different economies can eventually converge to their different steady-state levels.

#### 3.4.1. The *σ* Convergence Test

The logarithmic standard deviation of GTFP was chosen to reflect the regional variability of GTFP. If the standard deviation gradually decreases over time, it indicates that the variability between regions is getting smaller with a convergence trend. The formula is as follows:

(11) $$\sigma_{t}=\sqrt{\sum_{i=1}^{n}{\left(lnGTFP_{i,t}-\bar{\ln GTFP_{t}}\right)}^{2}/(n-1)}$$

**Figure 8:**
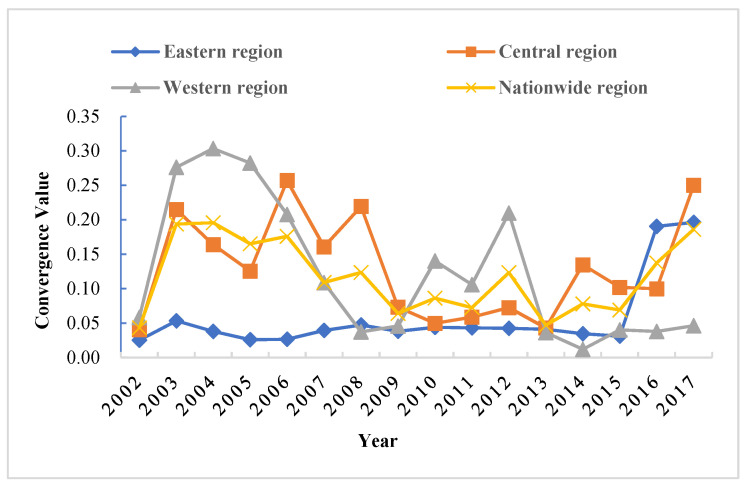
Evolution of *σ* convergence across the country and in the eastern, central, and western regions.

Table 6 and Figure 8 show that σ convergence existed in the standard deviation of GTFP in the western region throughout the study period, while no *σ* convergence was observed in the nationwide, eastern, and central regions. Figure 7 demonstrates that the variability of GTFP in the western region gradually disappeared over time, while the variability of GTFP in the nationwide, eastern, and central regions persisted and displayed a dispersion trend after 2014. The cross-sectional comparison reveals that among the three major groups, the mean of GTFP standard deviation was the largest in the central region (0.1084), followed by the western region (0.0830), and the smallest in the eastern region (0.0454), indicating that the internal regional variability of GTFP was more significant in the central region than in the eastern and western regions.

#### 3.4.2. Absolute *β* Convergence Test

The absolute *β* convergence model was obtained by applying the natural logarithm, calculated as follows.

(12) $$\ln(GTFP_{i,t+1}/GTFP_{i,t})=\alpha +\beta ln(GTFP_{i,t})+u_{i,t},\;$$

where $GTFP_{i,t}$ and $GTFP_{i,t+1}$ denote the GTFP growth rate at the beginning and end of a time period for region *i*, respectively. *i* denotes the *i*th region. *t* is the base year of a time period. *t* + 1 is the end year of a time period. $\ln(GTFP_{i,t+1}/GTFP_{i,t})$ refers to the average GTFP growth level of the *i*th region. The intercept terms are represented by α, and *β* denotes the growth rate coefficient. The random error term is $\mu_{i,t}$. Negative *β* indicates that absolute convergence is present.

Hausman test was conducted to determine the specific model and the overall significance of the fitted regression equation. The absolute *β* convergence of inter-provincial GTFP in China are shown in Table 7. The fixed-effects model was used to determine the overall significance of the regression equation for the nationwide and eastern regions, while the stochastic model was used to determine the overall significance of the regression equation for the central and western regions. The corresponding *β* values were negative for all regions, indicating that the absolute *β* convergence existed at the same level for all regions in terms of GTFP growth rate. The results revealed that the provinces across the country grew relatively synchronously with respect to GTFP, with internal differences gradually disappearing as time advanced. From a regional perspective, the estimated coefficients of all three regions were negative and significant, indicating that the gap in GTFP growth within the same region was gradually narrowing and that provinces within each region can achieve relatively synchronized development in GTFP. Club convergence existed in the western region due to the simultaneous presence of *σ* convergence.

#### 3.4.3. Conditional *β* Convergence Test

The following four control variables affecting GTFP were selected to be added to the absolute *β* convergence model, which was constructed as follows:

(13) $$\begin{array}{c} \ln(GTFP_{i,t+1}/GTFP_{i,t})=\alpha +\beta ln(GTFP_{i,t})+\beta_{1}Industry_{it}+\beta_{2}Finance_{it}\\ +\beta_{3}Open_{it}+\beta_{4}Energy_{it}+u_{i,t} \end{array}$$

The results presented in Table 8 show that the conditional *β* convergence coefficient was significantly negative in the eastern, central, and western regions as well as nationwide, indicating that the conditional convergence of GTFP was significant and that GTFP in the three regions would converge to their respective steady-state levels over time. The coefficient of the industrial structure was significantly positive in the eastern region and the whole country, revealing that the tertiary industry had a higher output value. The coefficient of fiscal concentration was significantly negative in the western region but not significant in the nationwide, central, and eastern regions, suggesting that all regions of the country need to improve the level of GTFP through the rational allocation of financial expenditure. The coefficient of opening-up degree was significantly negative in the western region but not in the nationwide, central, and eastern regions. Hence, China should continuously improve the level of openness to have a positive impact on enhancing GTFP. The coefficient of energy consumption structure was not significant at national and regional levels but was negative in the eastern and nationwide regions and positive in the central and western regions, showing that GTFP of central and western regions can be boosted by increasing the share of natural gas consumption.

## 4. Conclusions and Policy Implications

This study measured the GTFP of China in detail by constructing a meta-frontier and a group frontier function to eliminate the effect of regional differences on GTFP. The findings are as follows.

1. China’s inter-provincial green development efficiency varied significantly under the group frontier and meta-frontier. Under the meta-frontier, the mean values of green development efficiency during 2001–2017 were eastern region > central region > western region, while under the group frontier, the mean values were central region > western region > eastern region. Based on the technological gap ratio, the eastern region was closer to the meta-frontier in terms of green development efficiency technology, while the western and central regions were far away from the meta-frontier. This shows that the green development efficiency is preferable under the group frontier. Moreover, it also indicates the rationality and necessity of analyzing according to the three major groups.
2. China’s inter-provincial GTFP was measured based on the group frontier. The average value of GTFP in 30 provinces (municipalities and autonomous regions) was 1.043, and GTFP gradually increased. From a regional perspective, the three major regions of China showed large differences, with the western region maintaining the same trend as the whole country, the eastern region having a relatively stable development of GTFP as a whole, and the central region having an apparent upward trend of GTFP.
3. During the study period, the GTFP of 30 provinces (municipalities and autonomous regions) in China was relatively spatially stable, with the center of gravity shifting in the southwest–northeast direction. The range of the standard deviational ellipse showed a gradual decrease trend at each characteristic time point, indicating that the spatial distribution pattern of GTFP among Chinese provinces tended to be concentrated and relatively stable.
4. From the convergence test results, *σ* convergence existed only in China’s western region, and absolute *β* convergence and conditional *β* convergence were present in the whole country and in the eastern, central, and western regions. In terms of influencing factors, industrial structure and fiscal concentration had significant integrity. The industrial structure had a significant impact on the improvement of GTFP in the eastern region. Moreover, it is necessary for the central and western regions to accelerate the degree of market opening and the share of natural gas consumption, to enhance GTFP further.

Based on the findings above, we provide the following policy implications:

1. It is crucial to improve and develop the market-based environmental regulation system and it is necessary to solve the prominent problems in the trading of emission rights, carbon emissions, and water rights. In addition, it is also necessary to break down administrative divisions, scientifically allocate the total amount of pollutants in the region, and formulate corresponding incentives and penalties. Furthermore, different regions should accelerate the implementation of paid use system of resources, optimize the industrial structure through rationalizing environmental regulation policies, and thus promote the development of green industries.
2. It is essential to implement an innovation-driven strategy to enhance the value creation of factor resources, optimize factor allocation, and transform the mode of economic development. In addition, we can promote the effective flow of innovative factors and resources, adhere to scientific and technological innovation and institutional innovation, give full play to the advantages of dense innovative resources, and form an innovative agglomeration effect. It is also crucial to promote the development of a circular low-carbon economy by strengthening green technological innovation (green roofs, green facades, and carbon neutralization technology, etc.), promoting the concept of green ecological civilization, and achieving a win–win development model of environmental protection and economic growth.
3. It is necessary to implement regionally differentiated environmental regulation policies. The government should increase the ability to enhance green technology innovation in the central and western regions, establish a sound science and technology innovation system, and enhance the overall intensification of green resources within and between regions. It is also important for the government to build a regional joint prevention mechanism, promote joint innovation between regional industries, universities, and research institutes, protect the environment according to local conditions, and form a joint force to control pollution emissions. In this way, coordinated and green development can be promoted in cities located in different economic circles.
4. The legalization of environmental management and public participation in environmental protection needs to be strengthened. It is important to actively carry out various educational and publicity activities and promote energy-saving and emission-reducing consumption patterns and lifestyles. The work of education on the concept of ecological civilization requires the organic integration of the government, society, and schools. In addition, the government should improve the social supervision system, increase the transparency of public information, introduce the public supervision mechanism into the trading system of carbon emission rights and emission rights, and make public the trading information through various media such as newspapers, television, and the Internet to accept public supervision.
5. It is important to create a good macro policy and market environment, create a good institutional environment for green technology innovation, form an institutional mechanism conducive to the optimal allocation of science and technology resources, and realize the positive promotion effect of optimizing the system’s quality and improving GTFP. The government should promote its governance system and capacity, break down institutional barriers, and increase the value of factor resources for creativity. In this way, the economic construction and environmental protection can be mutually compatible to comprehensively increase China’s inter-provincial GTFP and promote China’s high-quality economic development.

## Figures and Tables

**Table 1:** Definitions and descriptive statistics for each indicator.

Targets	Unit	Minimum Value	Maximum Value	Average Value	(Statistics) Standard Deviation
Number of employed persons	10^4^ people	279.00	6767.00	2532.37	1684.78
Capital stock	CNY 10^8^	1562.06	199,488.71	32,069.38	31,840.33
Total energy consumption	10^4^ tons	520.00	38,899.00	11,396.82	7858.16
Gross domestic product (GDP)	CNY 10^8^	292.35	56,127.28	10,102.19	9928.89
Regional CO_2_ emissions	10^4^ tons	9.20	842.20	244.12	179.01
Industrial wastewater	10^4^ tons	3453.00	296,318.00	71,468.55	61,282.90
Industrial waste gas	10^8^ cubic meters	502.00	92,472.23	15,564.26	14,336.67
General industrial solid waste	10^4^ tons	75.00	45,576.00	7393.63	7419.01
*Industry*	%	29.70	80.60	42.22	8.48
*Finance*	%	7.72	62.69	20.17	9.21
*Open*	%	1.68	176.46	31.88	38.57
*Energy*	%	0.00	47.57	5.47	6.99

**Table 2:** China’s average inter-provincial green development efficiency and technology lag ratio under different frontiers.

Eastern Region	*Meta*	*Group*	*TGR*	Central Region	*Meta*	*Group*	*TGR*	Western Region	*Meta*	*Group*	*TGR*
Beijing	1.000	1.000	1.000	Shanxi	0.246	0.349	0.706	Chongqing	0.368	1.000	0.368
Tianjin	0.865	0.879	0.984	Inner Mongolia	0.328	0.961	0.341	Sichuan	0.390	1.000	0.390
Hebei	0.329	0.329	1.000	Jilin	0.392	0.668	0.586	Guizhou	0.215	0.570	0.378
Liaoning	0.509	0.520	0.979	Heilongjiang	0.720	1.000	0.720	Yunnan	0.307	0.813	0.378
Shanghai	1.000	1.000	1.000	Anhui	0.409	0.814	0.502	Shaanxi	0.310	0.773	0.401
Jiangsu	0.650	0.650	1.000	Jiangxi	0.418	0.844	0.495	Gansu	0.255	0.666	0.382
Zhejiang	0.591	0.591	1.000	Henan	0.413	0.633	0.651	Qinghai	0.217	0.508	0.426
Fujian	0.905	0.905	1.000	Hubei	0.329	1.000	0.495	Ningxia	0.154	0.330	0.469
Shandong	0.533	0.561	0.951	Hunan	0.497	0.972	0.511	Xinjiang	0.294	0.808	0.364
Guangdong	0.953	0.953	1.000								
Guangxi	0.362	0.362	1.000								
Hainan	0.699	0.699	1.000								
Average value	0.657	0.662	0.993	Average value	0.400	0.770	0.544	Average value	0.269	0.683	0.394

Note: *meta* denotes green development efficiency under meta-frontier. *group* denotes green development efficiency under group frontier. *TGR* denotes technological gap ratio.

**Table 3:** Green total factor productivity (GTFP) of provinces in China in some major years.

Provinces	2002	2008	2013	2017	Provinces	2002	2008	2013	2017
Beijing	1.089	1.174	1.079	1.099	Shandong	1.063	1.032	1.090	1.067
Tianjin	1.091	1.065	1.133	1.479	Henan	1.082	0.996	1.060	1.100
Hebei	1.051	1.014	1.014	1.032	Hubei	1.074	1.295	1.000	1.000
Shanxi	1.065	0.988	0.983	1.074	Hunan	1.100	1.611	1.109	1.123
Inner Mongolia	1.059	1.019	1.023	1.791	Guangdong	1.064	1.025	1.070	0.603
Liaoning	1.094	1.005	1.060	1.020	Guangxi	1.051	1.001	1.041	1.014
Jilin	1.062	1.004	1.087	1.007	Sichuan	1.107	1.000	1.000	1.000
Heilongjiang	1.162	1.000	1.000	1.000	Chongqing	1.149	1.061	1.000	1.000
Shanghai	1.082	1.023	1.027	1.000	Guizhou	1.057	1.069	1.003	0.990
Jiangsu	1.066	1.047	1.050	1.055	Yunnan	1.090	0.983	1.012	1.133
Zhejiang	1.053	1.057	1.042	1.012	Shaanxi	1.064	1.005	1.026	1.016
Anhui	1.105	0.947	1.030	1.039	Gansu	1.071	0.986	1.005	1.061
Fujian	1.042	1.035	1.054	1.206	Qinghai	1.096	1.069	0.991	1.017
Jiangxi	1.015	0.984	1.011	1.090	Ningxia	1.225	1.042	0.982	1.006
Hainan	1.005	0.999	0.968	0.972	Xinjiang	1.029	1.066	0.900	0.991

**Table 4:** Shifting direction and distance of center of gravity of inter-provincial GTFP in China.

Year	Center of Gravity Coordinates	Shifting Distance/km	Distance in East–West/km	Distance in North–South/km	Speed/(km/a)	East–West Speed/(km/a)	North–South Speed/(km/a)
2002	112.20° E						
	34.13° N						
2008	112.14° E	21.94	9.39	19.83	3.66	1.57	3.30
	33.95° N						
2013	112.44° E	30.27	26.23	15.11	6.05	5.25	3.02
	34.04° N						
2017	112.31° E	10.85	8.18	7.14	2.71	2.04	1.78
	34.45° N						

**Table 5:** Standard deviational ellipse parameters for the spatial distribution pattern of inter-provincial GTFP in China.

Year	Rotation Angle θ/°	Area/10^4^ km^2^	Standard Deviation along *x*-Axis/km	Standard Deviation along *y*-Axis/km	Shape Index
2002	44.139	384.484	1036.576	1180.737	0.878
2008	43.144	381.452	1045.959	1160.909	0.901
2013	40.891	376.723	1019.724	1176.014	0.867
2017	40.826	377.439	1029.901	1168.875	0.879

**Table 6:** *σ* convergence values for nationwide, eastern, central, and western regions.

Year	Eastern Region	Central Region	Western Region	Nationwide Region
2002	0.0252	0.0403	0.0583	0.0431
2003	0.0533	0.2147	0.2763	0.1937
2004	0.0379	0.1639	0.3033	0.1957
2005	0.0259	0.1256	0.2825	0.1652
2006	0.0265	0.2569	0.2077	0.1760
2007	0.0394	0.1607	0.1088	0.1090
2008	0.0473	0.2193	0.0372	0.1234
2009	0.0381	0.0731	0.0458	0.0640
2010	0.0440	0.0497	0.1403	0.0862
2011	0.0431	0.0587	0.1059	0.0726
2012	0.0425	0.0725	0.2097	0.1232
2013	0.0410	0.0428	0.0363	0.0468
2014	0.0346	0.1346	0.0119	0.0778
2015	0.0310	0.1019	0.0401	0.0690
2016	0.1905	0.0999	0.0380	0.1374
2017	0.1962	0.2499	0.0462	0.1861
Average value	0.0454	0.1084	0.0830	0.1045

**Table 7:** Inter-provincial GTFP absolute *β* convergence tests for China.

	Eastern Region	Central Region	Western Region	Nationwide Region
*β*	−1.599 ***	−1.289 ***	−1.196 ***	−1.276 ***
	(−15.408)	(−14.512)	(−14.194)	(−25.533)
Constant term	0.069 ***	0.039 ***	0.026 **	0.042 ***
	(10.345)	(3.311)	(2.049)	(7.184)
Model settings	fixed	random	random	fixed
Adj-R^2^	0.587	0.627	0.607	0.609
N	180	135	135	450
Conclusion	converge	converge	converge	converge

Note: ** *p* < 0.05, *** *p* < 0.01, *t*-values in parentheses.

**Table 8:** Inter-provincial GTFP conditional *β* convergence test for China.

	Eastern Region	Central Region	Western Region	Nationwide Region
*β*	−1.571 ***	−1.321 ***	−1.237 ***	−1.272 ***
	(−15.140)	(−14.691)	(−14.647)	(−26.166)
*β*_1_	0.003 ***	0.004	0.004	0.003 ***
	(4.467)	(1.441)	(1.354)	(3.725)
*β*_2_	−0.001	−0.003	−0.003 **	−0.002 **
	(−0.530)	(−0.946)	(−2.483)	(−2.421)
*β*_3_	0.000	−0.002	−0.004 *	−0.000
	(1.436)	(−1.044)	(−1.875)	(−0.510)
*β*_4_	−0.001	0.013	0.003	−0.000
	(−1.127)	(1.401)	(1.132)	(−0.089)
Constant term	−0.090 ***	−0.063	−0.024	−0.063 *
	(−3.611)	(−0.541)	(−0.186)	(−1.906)
Model settings	random	random	random	random
Adj-R^2^	0.606	0.636	0.631	0.618
N	180	135	135	450
Conclusion	converge	converge	converge	converge

Note: * *p <* 0.10, ** *p* < 0.05, *** *p* < 0.01. *t*-values in parentheses.

## Data Availability

The data presented in this study are available on request from the corresponding author. The data are not publicly available due to legal and privacy issues.
